# Analysis of the Excess of Papanicolaou Tests in Brazil from 2006 to 2015

**DOI:** 10.1055/s-0041-1741407

**Published:** 2022-01-29

**Authors:** Ana Carolina Pereira Fischer, Eduardo Augusto Pereira Fischer, Fernanda Brião Vaz, Júlia Hoffmann

**Affiliations:** 1 Department of Medicine, Universidade Regional de Blumenau, Blumenau, SC, Brazil; 2 Department of Medicine, Universidade Federal do Paraná, Curitiba, PR, Brazil

**Keywords:** Papanicolaou test, uterine cervix, cervical neoplasia, public health, teste de Papanicolau, colo do útero, neoplasias do colo, saúde pública

## Abstract

**Objective** To analyze the quantity of cervical smears, also designated Papanicolaou tests, between 2006 and 2015 in all the Federal units of Brazil, as well as to verify the quantity of exams collected outside the recommended age range and the economic impact of such excess.

**Methods** The data was collected from the Ministry of Health's database called *Sistema de Informação do Câncer do Colo de Útero* (SISCOLO), which contains all the test results collected nationwide by the Unified Health System (SUS, in the Portuguese acronym). From that, the number of exams and the age range of the women who underwent them were analyzed; besides, these numbers were stratified according to the state of where the exam was performed. The quantity of exams collected outside the recommended age range was verified, and, so, the economic impact generated was noted.

**Results** Between 2006 and 2015, 87,425,549 Papanicolaou tests were collected in Brazil. Of these, 20,215,052 tests were collected outside the age range recommended by the Brazilian Ministry of Health; this number corresponded to 23.12% of all exams. From such data, considering that each Pap smear collected by SUS generates a cost of BRL 7.30 to the government, according to the information in the *Tabela SUS* dated September 2018, there was a total charge of BRL 147,569,880 for tests collected outside the protocol.

**Conclusion** In Brazil, according to the Ministry of Health's protocol about the recommended practices on collecting Pap smears, whose newest edition dates of 2016, it is recommended that Pap smears are collected in women from a specific age range, in whom the potential diagnosing advantages overcome the onus of overdiagnosis or of a lesion with great regression potential. However, such protocols have not been correctly followed, promoting more than 20 million tests in excess, and an exorbitant cost for the Brazilian public health system. It is relevant to take measures to correctly use the official protocol, reducing the patients risks, as well as the economic impact for SUS.

## Resumo

**Objetivo** Analisar a quantidade de exames cérvico-vaginais, também chamados de Teste de Papanicolau, entre os anos de 2006 e 2015 em todos os estados brasileiros, bem como verificar o número de exames realizados fora da faixa etária indicada, e o impacto econômico desse excesso.

**Métodos** Os dados foram coletados a partir da base de dados do Ministério da Saúde chamada Sistema de Informação do Câncer do Colo de Útero (SISCOLO), que reúne os resultados de exames realizados em todo o Brasil pelo sistema único de saúde (SUS). A partir disso, foi analisado o número de exames e a faixa-etária de realização dos mesmos; além disso, esses números foram estratificados de acordo com o estado brasileiro de origem do exame. Foi verificada a quantidade de exames fora da idade recomendada, e, assim, foi observado o impacto econômico gerado.

**Resultado** Entre 2006 e 2015, 87.425.549 exames de Papanicolau foram realizados no Brasil. Deste montante, 20.215.052 testes foram realizados fora da faixa-etária preconizada pelo Ministério da Saúde do Brasil, o que equivale a 23,12% do total. A partir desse número, considerando que cada exame cérvico -vaginal realizado pelo SUS gera um custo de R$ 7,30 para o governo, de acordo com informações na Tabela SUS datada de setembro de 2018, foram gastos R$ 147.569.880 em exames realizados sem indicação.

**Conclusão** No Brasil, no protocolo do Ministério da Saúde sobre as práticas adequadas em coleta de exames cérvico-vaginais, sendo sua edição mais recente de 2016, a recomendação é realizar o teste de Papanicolau em mulheres dentro de uma faixa-etária específica, na qual a chance de se diagnosticar uma lesão supera o ônus de um sobrediagnóstico ou uma lesão com grande potencial de regressão. Entretanto, essa recomendação não tem sido seguida corretamente, gerando mais de 20 milhões de exames excedentes e um custo monetário exorbitante para o sistema público de saúde. É importante que medidas sejam tomadas para que o protocolo seja empregado corretamente a fim de reduzir riscos para a paciente, bem como a redução de gastos desnecessários para o SUS.

## Introduction

Cervical cancer is a public health issue, even though it is an avoidable disease. 1 2 It is the third most frequent tumor in women worldwide 2 and the fourth cause of death for malign tumors in women around the world, 1 responsible for the death of 274 thousand women every year worldwide. 3

In the past 30 years, a relationship between cervical cancer and human papillomavirus (HPV) has been established, justifying the disease's etiology. 4 Human papillomavirus infection may occur in up to 80% of women along their sexual life, 5 even though the infection alone is not sufficient for the cancer to evolve, so, mostly, the infection is transient and its natural course is spontaneous resolution, which can take from 6 months to 2 years. 6

Considering the natural history of the disease and its great regression potential, the guidelines from the Brazilian Ministry of Health recommend starting to collect Pap smears at 25 years-old, if the patient has already initiated her sexual life, repeating the exam annually for the first 2 years, and, from then on, if there is no cytological abnormality with malign potential, the collections may be performed every 3 years and should be interrupted when the woman reaches 64 years old. 7 There is a tendency of many women to do the Pap test annually, although it is known that reduction of the cumulative incidence of cervix invasion lesions is 93.5% when the test is done annually and 90.8% when done triennially, 8 which justifies the Brazilian guidelines. The excess of Pap smears, especially in those women who are not in the age range recommended by the Ministry of Health, overtaxes the public health system, especially considering that Brazil is a developing country and has a continent-dimension territory. Furthermore, it is important to consider quaternary prevention, which enforces the need to avoid possible damages associated with unnecessary medical interventions, such as iatrogeny.

Therefore, the objective of the present study is to verify the quantity of cervical screenings collected between 2006 to 2015 in all Brazilian states and the number of exams from 2006 to 2015 that did not fill the age criteria recommended by the Brazilian Ministry of Health, including its economic impact.

## Methods

This is a descriptive, transversal, and analytical study, and the data was collected from the Ministry of Health's online database called *Sistema de Informação do Câncer do Colo de Útero* (SISCOLO), which contains all the test results collected nationwide by the Unified Health System (SUS, in the Portuguese acronym). The target population of this study was women, especially those under 25 years-old as well as those older than 64 years old, who do not fill the recommended age range recommended by the Ministry of Health. From such data, we analyzed the quantity of exams performed between 2006 and 2015, in all Brazilian states, and how many were not done according to the Brazilian Ministry of Health protocol, whose guideline has been in place since 1986, and the conduct suggested by this guideline has been endorsed in every normative actualization about oncotic cytology, with the latest edition being from 2016. Moreover, also between 2006 and 2015, we analyzed the quantity of exams in each Brazilian region, and the economic impact of these surpluses, considering the rationalization and the proper management of public resources.

## Results

**Fig. 1:**
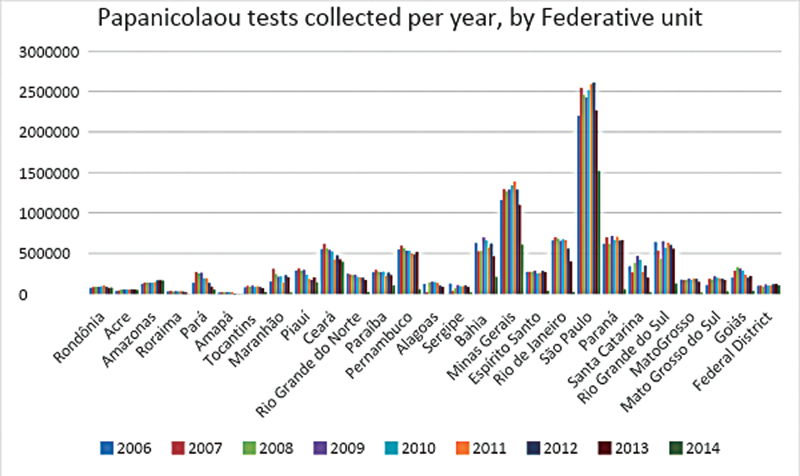
Papanicolaou tests collected per year, by Federative unit.

Based on the data collected from the SISCOLO platform, it became possible to accomplish a quantitative demographic analysis of age and geographic patterns of screening examinations on cervical cancer. In total, 87,425,549 Papanicolaou tests were collected throughout Brazil between 2006 and 2015. Such total number was stratified by age group and by Brazilian regions and states. Of that amount, 20,215,052 were made outside the Ministry of Health recommended age range, which consists in 23.12% of all collected exams during the evaluated period. It was observed that, considering absolute values, the Southeast region, in which the state of São Paulo stands out, had the highest number of exams during the period analyzed in this study, totalizing 39,801,111 Pap smears collected. Within that number, 9,166,999 of the Pap smears collected belong to women who are not in the age range recommended by the Brazilian Ministry of Health. The complete data about the number of Pap smears collected by year and by Federative unit is demonstrated in Fig. 1 .

**Fig. 2:**
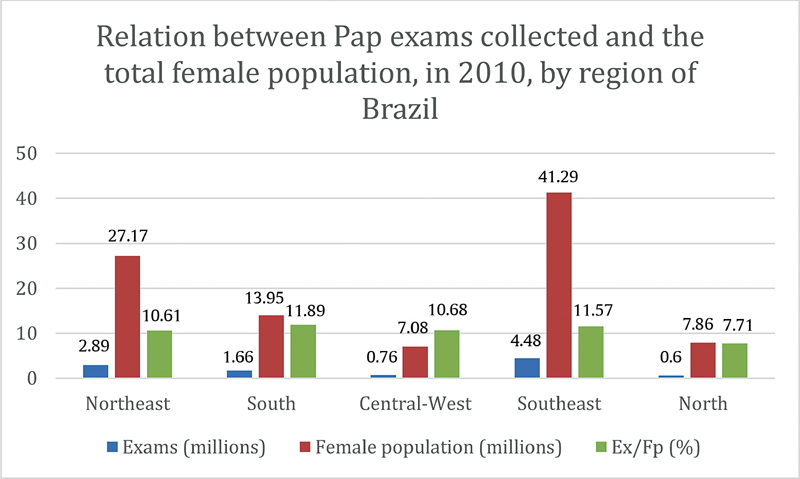
Relation between Pap exams collected and the total female population, in 2010, by region of Brazil.

It is relevant to highlight that, considering relative values, the Southeast region also has the lead, especially when the relation between the total number of Pap smears collected from 2006 to 2015 and the region's absolute female population in 2010 are compared, to use the median of the evaluated period. However, only in 2010, when the ratio between the total Pap smears collected in that year and the absolute female population are compared, the South region (11.89%), followed by Southeast region (11.57%), has the lead. It is relevant that, in the statistics related to the Pap tests collected in 2010 and its relationship with the total female population in that year, the maximum amplitude between the percentages in the South, Southeast, Central-West and Northeast, not including the North, was low (1.28 percentage point), which demonstrate some kind of uniformity in the exams collection, according to each region's population. The North region, on the other hand, showed the ratio of Pap smears collected in such year/female population in 2010 of 7.71% (0.0771), representing a result considerably below the national average (10.97%), while the Northeast (10.61%) and Central-West regions (10.68%), even though their statistics were also below the national average, showed smaller discrepancy. The detailed information about the relationship between the female population and the number of Pap smears collected in each region of Brazil can be found in Fig. 2 .

**Fig. 3:**
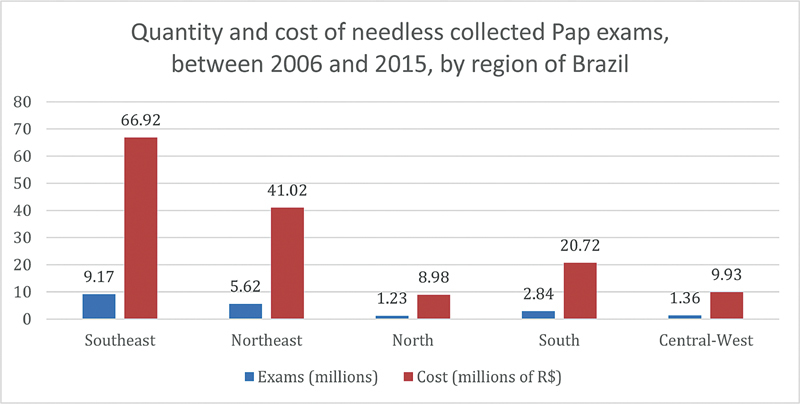
Quantity and cost of needless collected Pap exams between 2006 and 2015 by region of Brazil.

In another analysis, now comparing the total number of Pap smears collected between 2006 and 2015 and the female population in 2010, the North region also presented the lowest percentage among the 5 regions of Brazil (67.62%). Such percentage was below the national average too (88.53%), as well as the Central-West (84.81%) and Northeast (86.23%) regions, though such regions do not show as much discrepancy in relation to the national average as the North region does. Low population density, presence of many isolated territories and populations, as well as difficult access to medical care are factors that may explain such results. When analyzing the Pap tests collected between 2006 and 2015, it was observed that a relevant percentage of all exams were collected in women of the age range < 24 years old or > 65 years old, that is, out of the age gap recommended by the Brazilian Ministry of Health. More than 20% of all Pap smears were collected outside such protocol, reaching 23.98% in the Northeast region, and presenting the lowest rate in the South region, with 22.04%. Thus, there is a maximum amplitude among regions of 1.94 percentage point, while the national average is 23.12%. It is relevant, addressing economics aspects of such topic, account the cost to the public health system budget induced by the needless exams collected. Since the value spent by SUS for each Pap smear collected is BRL 7.30, information acquired by the *Tabela SUS* dated September 2018, in which the values paid to the service providers referred to the procedures covered by SUS are shown, it becomes possible to evaluate the excessive amount of money spent on collecting such unnecessary Pap smears. The total cost with unnecessary exams collected in a manner inconsistent with the protocol from the Ministry of Health all over Brazil during the cited period was BRL 147,569,880. The information regarding each Brazilian region is graphically represented in Fig. 3 .

## Discussion

Cervical cancer represents the third most common tumor in the Brazilian female population, and it is the fourth type of cancer in the cancer mortality scale in that same population. Still, especially in underdeveloped and developing countries, it is considered a public health issue and a major concern worldwide. 9

It is estimated that up to 80% of the sexually active population will acquire HPV over their lifetime. The article by de San José et al. 5 estimates that 291 millions of women have HPV and considers that 32% of them are infected by subtypes 16 and 18 , or both, which are known as high-grade oncogenics, and considering that, according to Ferlay et al., 10 the annual incidence, worldwide, is 530 thousand cervical cancer cases per year, it can be inferred that the presence of HPV alone is not a sufficient factor for the development of cancer, and it can be even considered a rare outcome.

Human papillomavirus infection is usually transient and has spontaneous regression, which may happen between 6 months and 2 years after acquiring the infection. 6 It is believed that low-grade squamous intraepithelial lesions (LSILs) do not actually represent a precursor lesion of cancer, but rather a cellular manifestation that denotes the virus infection, although high-grade squamous intraepithelial lesions have the potential to progress to cancer. 5

When there is infection by subtype 16 and it persists, it is estimated that the risk of developing cervical intraepithelial neoplasia grade 3 (CIN 3) or a more serious injury in 3 years is 5% and, in 10 years, 20%. 11

McCredie et al. 12 analyzed medical documents of a survey conducted in New Zealand between 1965 and 1974 in which patients with cervical cancer precursor lesions, in this case CIN 3, were not treated at the time, which was later considered unethical, and it was observed that the cumulative incidence of cervical cancer was 31.3% in 30 years, which corroborates the slow evolution of precursor lesions to cancer.

Recommendations of different societies worldwide are based on a study performed in 1986 by the International Agency of Research on Cancer, 13 which involved 8 countries and demonstrated that after a negative cervical cytology exam, in a 100% coverage population, there would be 93.5% reduction in the cumulative incidence of invasive cervical lesions in an annual screening, while in a triennial screening, the incidence reduction would be 90.8%. 8

The highest risk for cervical cancer is found in the age group between 45 and 49 years—that is, not in young women, with a newly initiated sexual life—and mortality increases with advancing age.

An American study performed by Watson et al. 14 evidenced that among 10,846 cases of cervical cancer diagnosed, 1.1% belonged to women up to 24 years old. Statistically, therefore, there is good reason, considering collective health, for the cytological examination of cervical mucous to be made years after the initiation of sexual life, and not as soon as a woman reaches adulthood or even earlier, unless there are significant factors to do so.

Therefore, it is important to implement quaternary prevention. It is composed by a group of actions that aim to reduce damage caused by unnecessary medical interventions. 15

The Center for Disease Control and Prevention (CDC), in its 2006 recommendations, 16 addresses the impact that the diagnosis of cancer precursor lesions may cause in teenagers. With this in mind, health attention to this group of women should instead emphasize the prevention of sexually transmitted diseases and contraception, considering that, at this age, the relationship between personal burden and preventative gain would be very high in favor of the burden. To confirm this statement, over 20% of the Papanicolaou tests made in Brazil between 2006 and 2015 were performed in women outside the age range recommended by the Ministry of Health patients were not between 25–64 years old). Given the fact that every Papanicolaou costs for the health system R$ 7.30 and considering the excessive number of unnecessary tests, its cost got to over R$ 147 million between the years of 2006 and 2015 in Brazil.

Vale et al. 17 measured, in two cities in the state of São Paulo, not only the number of smears collected outside the recommended age range, but also the number of exams collected biennial and annually, and not triennially, which is also contrary to the Brazilian Ministry of Health protocol. Such quantification promotes an accurate analysis of the real number, and proportion, of unnecessary smears collected. Such proportion, to give an example, has reached more than 60% of all Pap smears collected in the city of Amparo (SP) from 2001 to 2007. Therefore, an even bigger proportion than the one measured in this study considering only the age range.

The analysis by Van Ballegooijen et al. 18 of different screening patterns around the world, but specially in The Netherlands and Canada, demonstrated that, following some protocols about the beginning and the end of screening age, the interval between exams and population's coverage, it is possible to acquire less deaths and more years of life gained even with fewer collected smears and, thus, less unnecessarily treated women. Such results, according to the authors, might be obtained using efficient screening patterns, which focus on periodicity, longer intervals between exams, larger attendance, and rationalization of exam's age range.

Following the Ministry of Health protocol on Pap smears has benefits not only to the patient and to public health, but for the health budget too. Eliminating such waste in exams can cause considerable cost savings. In the USA, for example, it is estimated that, in 2011, between 158 and 226 billion dollars were spent in unnecessary tests, surgeries, medications, etc., 19 in which the unnecessary Papanicolaou tests are included.

## Conclusion

Considering the fact that cervical cancer is a public health issue, and it is the third most common tumor on women around the world, it is evident the importance of means for early detection of such lesions and to make a precise diagnosis. As seen in this study, there is an enormous amount (over 20 million) of tests performed in women who are not in the age range suggested by the Ministry of Health, corresponding to 23% of the total amount. Hence, it is necessary to question why those tests are performed in such a way in the whole Brazilian territory, even including women younger than 11 years old and older than 80 years old. Considering that there is a protocol by the Brazilian Ministry of Health, which is endorsed by scientific evidence recognized by organs such as World Health Organization and the American CDC, it would be expected that doctors are more cautious about requesting tests such as cervical screening for women who do not fill the age criteria recommended. Therefore, it is recommended that the Brazilian Ministry of Health and SUS watch carefully those numbers and values, in order for measures such as professional training, especially in Basic Units of Health, so changes could be seen in the way those exams are collected in Brazil. Furthermore, it is important to highlight that, in this article, there were not available information to know if those tests are being done every 3 years, accordingly to the Brazilian's health system protocol, or every 1 or 2 years, which means that the amount of unnecessary Pap smears is probably even greater than that calculated, because of the number of exams collected disregarding the correct time gap between exams. There are no similar studies comparing the cost of Pap smears and its impact on women's mortality or on the public health's budget. There are, however, other studies and estimates on the burden to the health system of excessive tests, surgeries, and medications, which are responsible for huge waste of public or private resources. This study's data represent an organization tool that allows the medical community to rethink its clinical practice, especially considering that in a continental-size country such as Brazil, minimal changes mean a lot of money spent. Hopefully, especially in Basic Units of Health, Pap smears will be collected according to t guidelines of the Brazilian Ministry of Health.
